# Anti-Inflammatory Potential of Novel Tethered Agonists of the Adhesion G Protein-Coupled Receptor F5

**DOI:** 10.3390/ijms27062648

**Published:** 2026-03-13

**Authors:** Artur Wnorowski, Diana Pietrzak-Mitura, Akanksha Mudgal, Lorenzo Scrofani, Magdalena Strachowska, Piotr Draczkowski, Krzysztof Jóźwiak, Jakub Fichna, Damian Jacenik

**Affiliations:** 1 Department of Biopharmacy, Faculty of Pharmacy, Medical University of Lublin, 20-059 Lublin, Poland; artur.wnorowski@umlub.pl (A.W.); 55923@student.umlub.pl (D.P.-M.); akanksha.mudgal@student.umlub.pl (A.M.); lorenzo.scrofani2@unibo.it (L.S.); krzysztof.jozwiak@umlub.pl (K.J.); 2 Department of Pharmacy and Biotechnology, University of Bologna, 40126 Bologna, Italy; 3 Department of Cytobiochemistry, Faculty of Biology and Environmental Protection, University of Lodz, 90-136 Lodz, Poland; magdalena.strachowska@biol.uni.lodz.pl; 4 Department of Synthesis and Chemical Technology of Pharmaceutical Substances, Faculty of Pharmacy, Medical University of Lublin, 20-059 Lublin, Poland; p.draczkowski@gmail.com; 5 Department of Biochemistry and Biophysics, National Bioinformatics Infrastructure Sweden, Science for Life Laboratory, Stockholm University, 114 19 Solna, Sweden; 6 Department of Biochemistry, Faculty of Medicine, Medical University of Lodz, 90-419 Lodz, Poland; jakub.fichna@umed.lodz.pl

**Keywords:** adhesion G protein-coupled receptor F5, ADGRF5, GPR116, tethered agonist, colitis, immune response

## Abstract

The adhesion G protein-coupled receptor F5 (ADGRF5) has been implicated in modulating immune responses in cancer; however, its role in inflammatory bowel diseases (IBDs), particularly colitis, remains largely unexplored. In this study, we aimed to design and characterize novel peptide agonists derived from the ADGRF5 *Stachel* sequence, as well as to evaluate their therapeutic potential in preclinical colitis models. *In silico* analysis and single amino acid substitutions within the ADGRF5 tethered agonist sequence, combined with functional assays in ADGRF5-overexpressing cells, including calcium mobilization and inositol phosphate production, were employed to assess the activity of novel ADGRF5 agonists. Western blot technique and murine model of colitis were used to evaluate downstream signaling pathways and immunomodulatory effects of ADGRF5 ligands. We identified a series of peptides exhibiting significantly enhanced ADGRF5 agonist activity, achieving up to a 6-fold increase in potency over the wild-type version. We identified critical substitutions within the *Stachel* sequence, namely S11N and D13S, as essential for improving agonistic activity. Finally, using these novel ADGRF5 agonists, we demonstrated their potent anti-inflammatory effects *in vivo*, showing that ADGRF5 activation ameliorates experimental colitis, as evidenced by reduced macroscopic damage scores and improved colon architecture. These findings establish ADGRF5 as a potential therapeutic target for colitis and highlight the promise of *Stachel*-derived peptide agonists for the development of novel anti-inflammatory therapies.

## 1. Introduction

**Figure 1:**
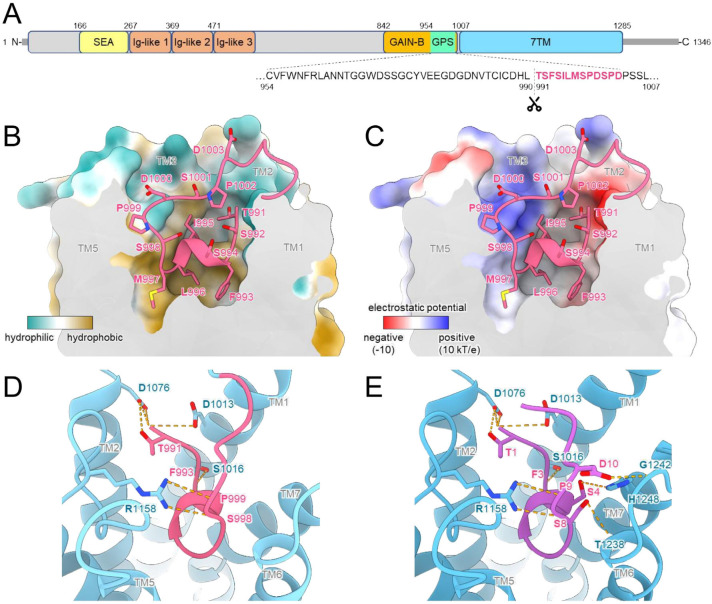
Predicted structural arrangement of the *Stachel* sequence within the ADGRF5 binding pocket. (**A**) Schematic representation of the full-length ADGRF5 domain arrangement, with an enlarged view of the GPS region containing the *Stachel* sequence (residues highlighted in pink correspond to those labeled in pink in panels (**B**–**D**) and the associated cleavage site indicated). (**B**) Cross-sectional view of the receptor structure along its transmembrane region (TM6 and TM7 not visible) illustrating the hydrophobic characteristics of the interaction between the *Stachel* core and the innermost part of the binding pocket. (**C**) The same view highlighting the polar nature of the interactions at the entrance of the ADGRF5 binding pocket. (**D**,**E**) Differences in hydrogen bonds formed between the ADGRF5 TM helices and the *Stachel* sequence bound as the tethered agonist (pink) or as free peptide (PL01^WT^—purple), respectively.

The adhesion G protein-coupled receptor F5 (ADGRF5; formerly known as GPR116) is implicated in a range of physiological and pathological processes, including lung surfactant homeostasis, immune response, glucose intolerance and insulin resistance, as well as cancer progression [1]. ADGRF5 belongs to the group of adhesion G protein-coupled receptors (GPCRs)—a unique class of 7-transmembrane (7TM) receptors featuring an extended N-terminal ectodomain composed of multiple motifs facilitating cell–cell and cell-matrix interactions [2]. As depicted in Figure 1A, in ADGRF5, the ectodomain contains immunoglobulin-like repeats and SEA domain [3,4]. The membrane-anchored C-terminal segment is linked to the N-terminal region through the GPCR autoproteolysis-inducing (GAIN) domain, which includes a highly conserved GPCR proteolysis site (GPS). Autoproteolytic cleavage at the GPS generates a tethered peptide agonist at the N-terminus of the cleaved receptor. This agonistic fragment, known as the *Stachel* sequence, is located between the GPS cleavage site and the first transmembrane (TM) helix. Following cleavage, the *Stachel* sequence remains attached to the receptor and acts as an intrinsic agonist [2] (Figure 1A–C).

Recent evidence indicates that synthetic peptides corresponding to the *Stachel* sequence can serve as external agonists of adhesion GPCRs, offering a promising approach to modulate ADGRF5 activity [5]. A study by Brown et al. documented that a peptide composed of at least 9 amino acids (aa) from the *Stachel* sequence is required for ADGRF5 activation [6]. This core region of the *Stachel* is highly conserved and essential for its agonistic activity, as confirmed by alanine-scanning experiments [6]. Further experiments demonstrated that a 13-aa long peptide, followed by a shorter 12-aa long peptide version, are optimal for ADGRF5 activation [5].

ADGRF5 activated by mature tethered agonist modulates heterotrimeric G proteins, such as Gα_q/11_, regulating inositol phosphate (IP) turnover and calcium mobilization [6,7]. These events are critical not only at the cellular level but ADGRF5-dependent signaling contributes to the progression of numerous human diseases [8,9,10,11,12,13]. For instance, emerging evidence suggests that ADGRF5 modulates an immune responses during tumorigenesis [1]. However, the significance of ADGRF5 in colitis, a condition characterized by dysregulated immune response, has been largely overlooked [14,15,16].

Here, we designed a series of novel *Stachel*-derived ADGRF5 agonists that exhibited up to 6-fold higher potency than the wild-type peptide. Then, we exploited these optimized synthetic peptides to reveal the anti-inflammatory role of ADGRF5 in the murine model of colitis. Observed anti-inflammatory effects indicate that ADGRF5 is a promising therapeutic target for IBDs.

## 2. Results

### 2.1. C-Terminal Region of Stachel Peptide Is Crucial for ADGRF5 Activity

To understand the mechanism by which the *Stachel* sequence activates ADGRF5, we performed structural modeling of the receptor with its tethered agonist using AlphaFold3 (AF3) [17]. AF3 consistently predicted the binding of the *Stachel* sequence within a pocket formed by TM1, TM2, TM3, TM5, TM6, and TM7 of ADGRF5, with high confidence; best model showed average per residue confidence prediction (pLDDT) = 73.06 and overall accuracy of the predicted structure (pTM) = 0.76. The predicted binding mechanism exploited the amphipathic nature of the *Stachel* peptide. Within the receptor pocket, the *Stachel* peptide adopted a U-shaped conformation. Its central, predominantly lipophilic region, composed of phenylalanine, serine, isoleucine, leucine, and methionine residues, was deeply inserted into the binding pocket. In contrast, the polar residues at both termini of the core *Stachel* peptide, and the linker region connecting the *Stachel* peptide to TM1, remained closer to the receptor surface and largely solvent-exposed. This spatial arrangement was consistent with the chemical environment of the binding pocket, which is characterized by a predominantly hydrophobic interior, lined with lipophilic residues, and a more polar entrance (Figure 1B). Notably, the C-terminal region of the tethered *Stachel* peptide, which connects it to the TM domain of ADGRF5, did not contribute to the interaction, likely due to its limited conformational freedom. To further investigate the potential role of this C-terminal region, we modeled the interaction of ADGRF5 with an unbound peptide, PL01^WT^ (residues 991-1003), encompassing the wild-type *Stachel* sequence. This free peptide would possess greater conformational freedom at its C-terminus. AF3-predicted models of this complex revealed that the exogenous peptide, PL01^WT^, displaced the endogenous tethered agonist. In this configuration, Asp10 and Ser8 at the C-terminus of PL01^WT^ formed additional hydrogen bonds with the receptor (Figure 1E). The flexibility of the C-terminus of the peptide accompanied by a conformational change in TM1 and TM7 of the receptor facilitated formation of a hydrogen bond between Ser4 in the *Stachel* peptide and His1248 in TM7 while enabling Pro12 to form new interactions with the hydrophobic interface between the TM1 and TM7. These observations suggest that the C-terminal region of the *Stachel* peptide has the capacity to contribute to the interaction with ADGRF5, and its further optimization could lead to enhanced binding affinity.

### 2.2. Modifications at Positions 11 and 13 Improve the Functional Potency of the Stachel-Derived Peptide

**Figure 2:**
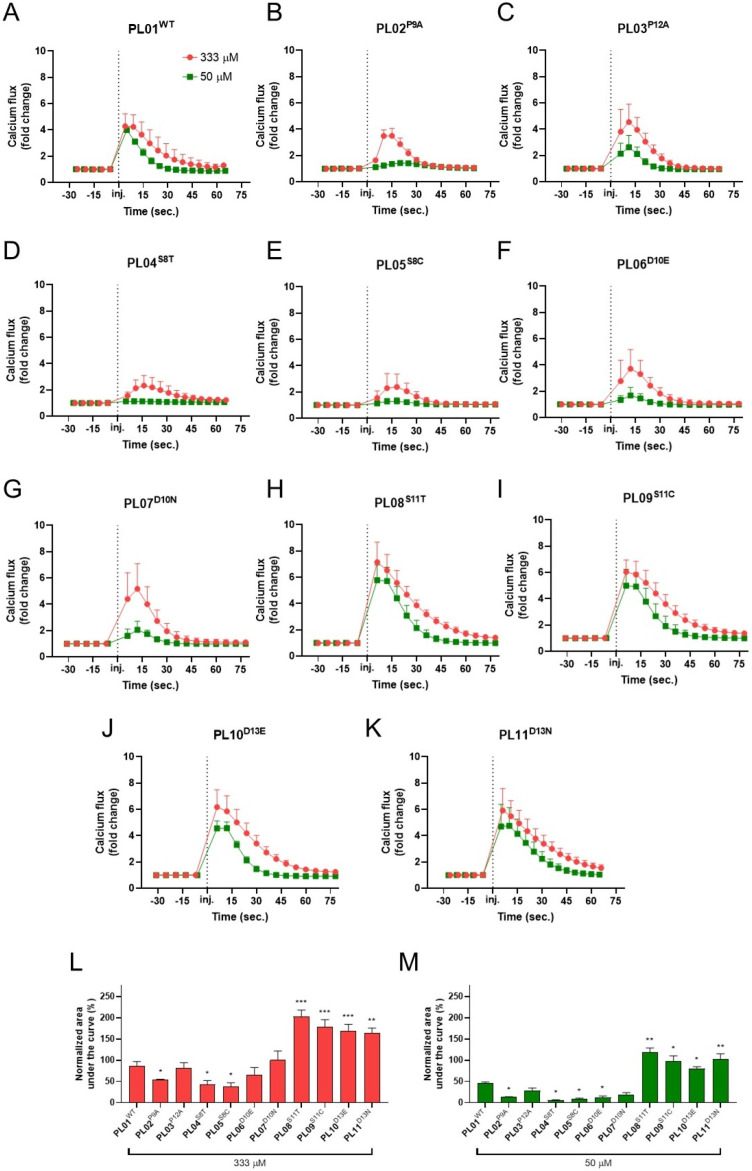
Identification of crucial amino acids in the sequence of tethered ADGRF5 agonists responsible for their activity. Activity of mature tethered ADGRF5 agonist (**A**) and peptides with modification in positions 8, 9, 10, 11, 12, and 13 (**B**–**K**) were estimated using calcium flux and compared to mature tethered ADGRF5 agonist (**L**,**M**). To note, red color represents high concentration (333 µM) and green color represents low concentration (50 µM); the dotted line marks injection timepoint of the peptides. Data are expressed as mean ± SEM, n = 3 for independent experiments and n = 3–4 for technical replicates. * *p* < 0.05, ** *p* < 0.01, *** *p* < 0.001 vs. P001.

Building upon these structural insights, we next experimentally investigated the potential for optimizing the C-terminal region of the endogenous ligand to enhance its activity at ADGRF5. For this purpose, we employed a 13-aa peptide, PL01^WT^, corresponding to the wild-type ADGRF5 *Stachel* sequence, as a representative agonist. As illustrated in Figure 2A, PL01^WT^ effectively activated ADGRF5 in HEK-293 cells with ADGRF5 overexpression (HEK-ADGRF5), inducing rapid calcium accumulation in a dose-dependent manner. Of note, we also used 13-aa peptide corresponding to the ADGRF4 *Stachel* sequence described as negative control peptide (NCP, Table 1) and, as we documented, the above-mentioned peptide was unable to activate ADGRF5 (Supplementary Figure S1). Specifically, robust calcium influx was observed at both a high concentration (333 µM) and a lower concentration (50 µM) of PL01^WT^, demonstrating the agonist potential of this peptide. A recent study using alanine-scanning mutagenesis indicated that a minimum of 9 aa from the *Stachel* sequence are necessary for ADGRF5 activation [18]. Additionally, Pro residues within the *Stachel* sequence were postulated to be particularly important for tethered peptide activity. To verify that hypothesis, we generated PL01^WT^ variants in which Pro residues at position 9 or 12 were individually substituted with Ala (PL02^P9A^ and PL03^P12A^, respectively; Table 1). As depicted in Figure 2A–C, both PL02^P9A^ and PL03^P12A^ were capable of inducing calcium accumulation in HEK-ADGRF5 cells. However, their potency was significantly reduced compared to PL01^WT^, suggesting that both Pro9 and 12 are both required for the optimal activity of the *Stachel*-derived peptide. This aligns with the predicted structural details of the *Stachel*-ADGRF5 interaction, in which Pro residues were inducing kinks crucial for adapting U-shape conformation; additionally, Pro9 was found wedged between TM5 and TM7, reducing their mobility or inducing particular conformations (Figure 1B–E). Previous studies by Bridges et al. identified that Phe, Leu and Met residues at positions 3, 6 and 7, respectively, as critical residues for ADGRF5 agonist activity [19]. As modeled in the current study, these residues, together with Ile5, compose the lipophilic region in the *Stachel* sequence that, upon binding, gets buried within the receptor’s pocket. Consequently, they appear crucial for an energetically favorable entropic effect of the binding that redistributes ordered water molecules from the receptor hydrophobic pocket and around the peptide apolar residues back into bulk solution. Therefore, we refrained from modifying the hydrophobic core region and focused our subsequent modifications on the C-terminal tail of the *Stachel*-derived agonist. Specifically, we introduced substitutions at positions 8, 10, 11, or 13, replacing the native Ser and Asp residues with aa of similar properties, Thr or Cys as well as Glu or Asn, respectively (Table 1). The peptides modified at positions 11 or 13, but not at positions 8 or 10, elicited significantly enhanced calcium influx (Figure 2D–K). These data indicate that the ADGRF5 agonists PL08^S11T^, PL09^S11C^, PL10^D13E^, and PL11^D13N^ exhibit improved ADGRF5 activation potential compared to PL01^WT^. This is consistent with findings from Brow et al., who noted that Asp in position 10 is critical for the interaction of the *Stachel*-derived agonist with the receptor, as peptide truncation to just 9 aa markedly decreased IP conversion in cells overexpressing ADGRF5 [6]. In general, our findings extend the observations by Brow et al., demonstrating that Ser at position 8, and not just Pro residues at positions 9 and 12 and potentially Asp at position 10, is important for the optimal activity of ADGRF5 tethered agonists. Collectively, the comparative analysis of wild-type and modified ADGRF5 agonist activity revealed that modifications at positions 11 and 13 can enhance the functional potency of the *Stachel*-derived peptide (Figure 2K and L, respectively).

### 2.3. Identification of Novel Agonists with Enhanced Potency to Activate ADGRF5

**Figure 3:**
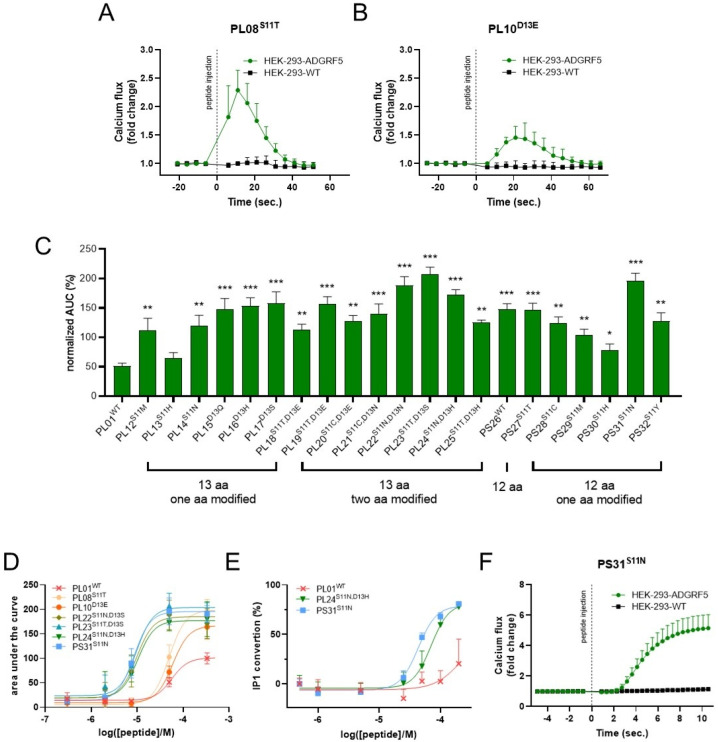
Identification of amino acids in positions 11 and 13 in the sequence of tethered ADGRF5 agonists critical for their activity. Activity of PL8^S11T^ peptide (**A**) and PL10^D13E^ peptide (**B**) in HEK-293 cells and HEK-293 cells with ADGRF5 overexpression was estimated by calcium flux. Activity of mature tethered ADGRF5 agonist (**A**) and peptides composed of 12 and 13 amino acids corresponding to *Stachel* sequence with modification in positions 11 and 13 (**C**) was estimated using calcium flux and compared to mature tethered ADGRF5 agonist. The value of EC_50_ for PL01^WT^, PL08^S11T^, PL10^D13E^, PL22^S11N,D13S^, PL23S^11T,D13S^, PL24^S11N,D13H^ and PS31^S11N^ peptide estimated by calcium flux (**D**) and IP conversion (**E**) assays in ADGRF5 stably expressing HEK-293 cells. Activity of PS31^S11N^ peptide (**F**) in HEK-293 cells and HEK-293 cells with ADGRF5 overexpression was estimated by calcium flux. Data are expressed as mean ± SEM, n = 3 for independent experiments and n = 3–4 for technical replicates. * *p* < 0.05, ** *p* < 0.01, *** *p* < 0.001 vs. P001.

Next, we validated the specificity of the observed calcium flux using the optimized ADGRF5 agonists, PL08^S11T^ and PL10^D13E^. As demonstrated in Figure 3A,D, both PL08^S11T^ and PL10^D13E^ effectively triggered calcium influx specifically in HEK-ADGRF5 cells, but not in control wild-type HEK-293 cells. Subsequently, we explored further modifications at positions 11 and 13, generating a diverse panel of analogs bearing single and double aa substitutions (Table 1). In addition, we evaluated the activity of a truncated peptide, PS26^WT^, representing a 12-aa fragment of the native ADGRF5 *Stachel* sequence (Table 1). Remarkably, as illustrated in Figure 3C, these additional modifications significantly enhanced the agonist response. When screened at a fixed concentration, several optimized peptides induced up to a 4-fold greater response magnitude (as measured by calcium flux) compared to PL010^WT^ at the same concentration.

To establish the dose–response relationships and quantify the potency of our optimized agonists, we next determined the EC_50_ values for a selection of *Stachel*-derived agonists, namely PL01^WT^, PL08^S11T^, PL10^D13E^, PL22^S11N,D13S^, PL23^S11T,D13S^, PL24^S11N,D13H^, and PS31^S11N^, using both calcium accumulation (Figure 3D) and inositol phosphate (IP) conversion assays (Figure 3E). As illustrated in Figure 3D, PL01^WT^, PL08^S11T^ and PL10^D13E^ activated ADGRF5 with EC_50_ values of 59.4, 56.4, and 49.7 µM, respectively. Interestingly, peptides incorporating combined modifications (PL22^S11N,D13S^, PL23^S11T,D13S^, PL24^S11N,D13H^) and the truncated, single-substitution analog PS31^S11N^ showed markedly lower EC_50_ values of 9.7, 9.6, 10.4, and 8.5 µM, respectively (Figure 3D). This trend was further corroborated in the IP accumulation assay, where PL01^WT^ activated ADGRF5 with EC_50_ value of 219.5 µM, but PL24^S11N,D13H^ and PS31^S11N^ generated the half-maximal activation at doses as low as 70.4 and 43.4 µM, respectively (Figure 3E). Additionally, we confirmed that Ca^2+^ accumulation elicited by PS26^WT^ was due to ADGRF5 activation as the effect was absent in wild-type HEK-293 cells (Figure 3F), reinforcing its receptor specificity of the truncated, 12-aa peptide. Taken together, these findings demonstrate the successful development of ADGRF5 agonists with up to a 6-fold enhancement in potency (as evident by the decrease in the EC_50_ values) in comparison to the parental peptide, PL01^WT^.

### 2.4. ADGRF5 Agonists with Modified Stachel Sequence Trigger Intracellular Signaling

**Figure 4:**
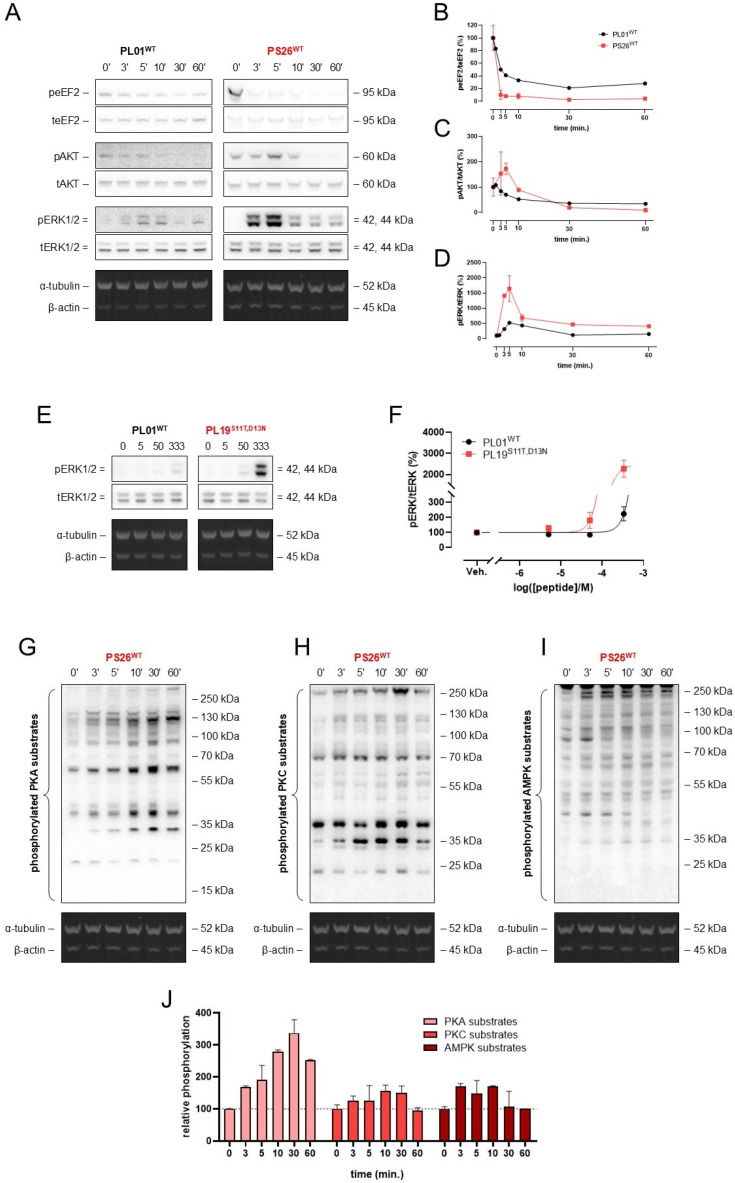
The effect of ADGRF5 activation using PS26^WT^ peptide on cellular signaling. The representative images and quantitative ratio obtained from Western blot analysis of non-activated and activated form of EF2, AKT, and ERK1/2 (**A**–**D**) in HEK-293 cells with ADGRF5 overexpression under PS26^WT^ stimulation in relation PL01^WT^ peptide. The representative images (**E**) and quantitative ratio (**F**) obtained from Western blot analysis of non-activated and activated forms of ERK1/2 in HEK-293 cells with ADGRF5 overexpression under PL19^S11T,D13N^ stimulation in relation to PL01^WT^ peptide. The representative images (**G**–**I**) and quantitative ratio (**J**) obtained from Western blot analysis of phosphorylated PKA (**G**), PKC (**H**), and AMPK (**I**) substrates in HEK-293 cells with ADGRF5 overexpression under PS26^WT^ stimulation. To note, the dotted line represents the level of PKA, PKC, and AMPK phosphorylation in untreated cells. Data are expressed as mean ± SEM; n = 3 for independent experiments and n = 3–4 for technical replicates.

To explore the ability of our novel ADGRF5 agonists to induce downstream signaling, we incubated HEK-ADGRF5 cells with PL01^WT^, PS26^WT^, and PL19^S11T,D13N^. As shown in Figure 4A–D, PS26^WT^ induced the time-dependent phosphorylation of AKT and ERK1/2, but it did not affect the phosphorylation of eukaryotic elongation factor 2 (eEF2). Notably, we observed higher levels of phospho-ERK1/2 in HEK-ADGRF5 cells treated with PL19^S11T,D13N^ compared to cells treated with PL01^WT^ (Figure 4E,F). Moreover, incubation of HEK-ADGRF5 cells with PS26^WT^ resulted in well-pronounced time-dependent phosphorylation of protein kinase A (PKA) substrates, but with little effect on the substrates of protein kinase C (PKC) nor on those of 5′ AMP-activated protein kinase (AMPK) (Figure 4G,J). Overall, this finding documented that ADGRF5 agonists with modified *Stachel* sequence enhance intracellular signaling when compared to the wild-type 13-aa peptide.

### 2.5. ADGRF5 Agonists with Modified Stachel Sequence Prevent Colitis Progression

**Figure 5:**
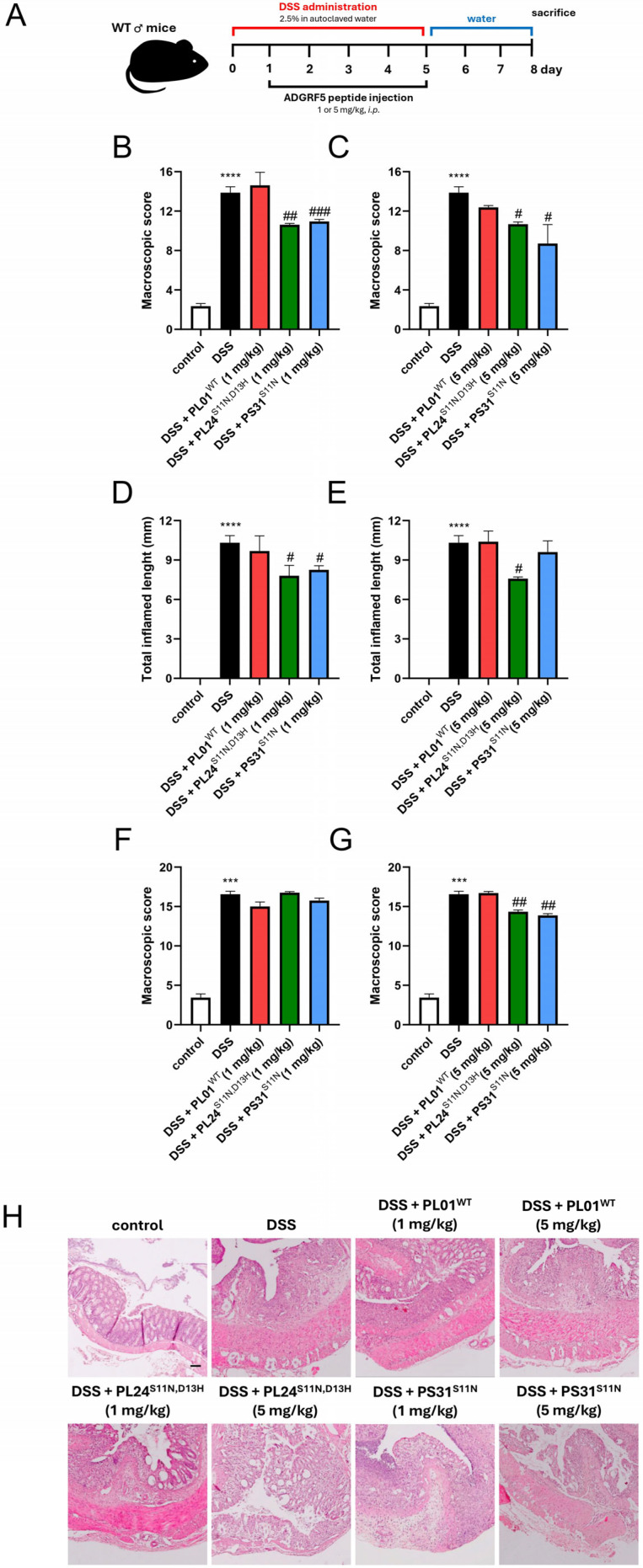
The tethered ADGRF5 agonists and their anti-inflammatory action in the murine model of colitis. (**A**) The scheme representing timeline for induction of murine model of colitis and tethered ADGRF5 agonist treatments. The macroscopic score (**B**,**C**), total inflammation length (**D**,**E**), and the microscopic score (**F**,**G**) in control mice and untreated mice with colitis as well as mice with colitis under PL01^WT^, PL24^S11N,D13H^ and PS31^S11N^ treatment at doses of 1 mg/kg and 5 mg/kg. Representative images of hematoxylin and eosin staining of colon cross-section (**H**) obtained from control, DSS, and treated groups. Data are expressed as mean ± SEM, with n = 10–12 mice per group. *** *p* < 0.001, **** *p* < 0.0001 vs. control, # *p* < 0.05, ## *p* < 0.01, ### *p* < 0.001 vs. DSS. Scale bar—100 µm.

Accumulating data suggests that ADGRF5 plays an important role in the immune response, which was highlighted mainly in the respiratory tract [1,9,11,19]. In fact, studies employing *ADGRF5*-deficient mice indicated that ADGRF5 is crucial for surfactant homeostasis, and its loss is associated with inflammation. Moreover, a body of evidence highlighted ADGRF5 function in the context of cancer immunity [14,15,16]. For instance, a recent study conducted by Guo et al. linked ADGRF5 knockout in natural killer cells with their improved anti-tumor activity in pancreatic cancer [14]. Nevertheless, the role of ADGRF5 in colitis remains poorly understood. Based on available evidence, we hypothesized that activation of ADGRF5 may suppress the immune response in the gastrointestinal tract. To explore the immunomodulatory potential of our novel ADGRF5 agonists *in vivo*, we administered PL01^WT^, PL24^S11N,D13H^ and PS31^S11N^ to mice with dextran sulfate sodium (DSS)-induced colitis (Figure 5A). PL24^S11N,D13H^ (13-aa peptide) and PS31^S11N^ (12-aa peptide), both derived from the ADGRF5 *Stachel* sequence, were selected based on their enhanced *in vitro* activity; PL01^WT^ was included as a reference. As shown in Figure 5A, colitis was induced by DSS administration from day 0 to day 5 while ADGRF5 agonists were injected intraperitoneally (i.p.) once daily from day 1 to day 5 at a dose of 1 mg/kg or 5 mg/kg. DSS administration significantly increased the macroscopic score, total inflamed length, and microscopic score compared to control mice (Figure 5B,C,E). The analysis of colons from each treatment group revealed that modified ADGRF5 agonists, but not PL01^WT^, improved macroscopic parameters (Figure 5B). Notably, ineffectiveness of PL01^WT^ in resolving colitis in mice seems to be related to its significantly lower activity against ADGRF5, as demonstrated in our *in vitro* studies. This limited activity may be exacerbated by its susceptibility to proteolysis within a living system. This, combined with relatively low activity of PL01^WT^, may prevent the generation of a sufficient signal to elicit anti-inflammatory responses. In contrast, a statistically significant reduction in total inflamed length was observed after administration of PL24^S11N,D13H^ at a dose of 5 mg/kg and PS31^S11N^ at both 1 mg/kg and 5 mg/kg (Figure 5C). Moreover, histological analysis revealed that administration of both PL24^S11N,D13H^ and PS31^S11N^ at 5 mg/kg resulted in a marked improvement in colon architecture (Figure 5D,E).

## 3. Discussion

Inflammatory bowel diseases (IBDs) are chronic disorders of the colon characterized by an overactive immune response against an unknown factor. The pathophysiology of Crohn’s disease (CD) and ulcerative colitis (UC)—the two main entities of IBDs—is complex and involves, among other factors, genetic variation, immune dysregulation, and environmental influences. The global prevalence of IBDs is rising, and clinicians and patients with CD and UC still face limited therapeutic options to achieve sustained resolution of colitis, especially during the acute phase of these diseases. In fact, endoscopic remission rates in CD patients receiving adalimumab, infliximab, ustekinumab, or vedolizumab were found to be 52%, 53%, 56%, and 51%, respectively [20]. The efficacy of biological treatment, which is currently the most effective approach for patients with IBDs, still falls below expectations. New and more effective strategies must be developed to maintain long-term remission in colitis and improve patients’ quality of life.

Here, we identified ADGRF5 as a novel and potent modulator of colitis. ADGRF5 is a member of the adhesion GPCR family, and its significance has been described for many physiological processes such as lipogenesis, glucose and insulin metabolism, renal acid secretion, and the synthesis and secretion of pulmonary surfactant [1]. The expression and action of ADGRF5 appear to be crucial not only under normal conditions but also in numerous human diseases such as osteoporosis and neoplastic transformation, among others [1]. Recently, Ariestanti et al. found that ADGRF5 knockout mice developed emphysema [9]. The action of ADGRF5 was linked to the promotion of an immune response mediated by monocytes/macrophages, leading to hypersecretion of numerous pro-inflammatory cytokines and chemokines. Further evidence from cancer-focused research revealed an immunomodulatory role for ADGRF5, supporting the hypothesis regarding its immune-related function [14,15,16]. Based on the aforementioned reports, we postulated that ADGRF5 may affect not only the immune response in the lungs or during tumorigenesis but could also be involved in colitis progression.

To explore the significance of ADGRF5 in colitis, we designed and validated a new series of ADGRF5 peptide agonists, identifying numerous peptide agonists with significantly higher potency against ADGRF5 compared to the parental peptide, PL01^WT^. It is worth noting that four residues in the sequence of the ADGRF5 *Stachel* region, i.e., Ser8, Pro9, Asp10, and Pro12, are critical for the activity of ADGRF5-tethered agonists. Moreover, we demonstrated the specificity of the modified peptides using wild-type HEK-293 cells and HEK-293 cells overexpressing ADGRF5. Our findings show that ADGRF5 tethered agonists were able to induce downstream signaling pathways such as AKT and ERK1/2, demonstrating the functionality of the ADGRF5 peptide agonists. Finally, using a chemically induced murine model of colitis, we observed that administration of the ADGRF5 peptide agonist attenuated the progression of colitis at both macroscopic and microscopic levels. Despite the short half-life of the peptide *in vivo*, we were able to demonstrate that ADGRF5 agonists reduce colitis. Further modification of the ADGRF5 tethered agonists to extend their presence in the body may enhance their therapeutic effects.

Overall, our findings demonstrate that modified *Stachel*-derived ADGRF5 agonists possess anti-inflammatory properties and that ADGRF5 activation holds therapeutic potential for mitigating colitis. Moreover, the sequence of the ADGRF5 Stachel region, i.e., TSFSILMSPDSPD, is identical in mouse and human, which strongly suggests that the agonists and its analog-modified peptide agonists may prevent inflammation in patients with IBDs. We acknowledge that our study has limitations such as lack of mechanistic determination of ADGRF5-dependent signaling pathway in colitis, and further research is needed to elucidate the mechanisms underlying the anti-inflammatory action of these peptide agonists. However, to our knowledge, this is the first evidence supporting the targeting of ADGRF5 as a novel treatment modality for colitis management.

## 4. Materials and Methods

### 4.1. Prediction of the ADGRF5 Structure Using AlphaFold

The structural analysis of ADGRF5 interaction with the *Stachel* sequence was performed based on the structures predicted using AlphaFold3 Server [17]. The sequence of the ADGRF5 was retrieved from the UniProt database with the ID Q8IZF2. For the structure prediction, the N-terminal part of the full-length receptor sequence was truncated, leaving the region corresponding to residues 991–1346 of the full-length protein. Predictions were conducted with 5 different random seeds, yielding a total of 25 predicted models. The obtained structures were assessed for their quality based on the prediction metrics; pLDDT (per residue confidence prediction), pTM (overall accuracy of the predicted structure), and PAE plots (presenting error in the relative position of the structure elements). UCSF ChimeraX was used to visualize and analyze the predicted structures [21].

### 4.2. Cell Culture

HEK-293 cells and HEK-293 cells with ADGRF5 overexpression (HEK-ADGRF5) were obtained from American Tissue Culture Collection (Manassas, VA, USA) and Innoprot (Derio, Spain), respectively. HEK-293 cells were maintained in Dulbecco’s Modified Eagle’s Medium (DMEM) supplemented with 10% fetal bovine serum (FBS), 2 mM L-glutamine, 100 U/mL penicillin, and 0.1 mg/mL streptomycin. Cell culture medium for HEK-ADGRF5 cells were additionally supplemented with 80 µg/mL hygromycin and 1 × MEM non-essential amino acid solution. All culture media and supplements were from ThermoFisher Scientific (Waltham, MA, USA). Cells were maintained in a standard environment (37 °C under humidified 5% CO_2_ in air), and the medium was replaced every 2–3 days.

### 4.3. Characterization of HEK-293 Cells with ADGRF5 Overexpression

#### 4.3.1. Reverse Transcription Polymerase Chain Reaction

HEK-ADGRF5 cells were cultured in a 24-well plate and RNA was extracted using commercially available TRI Reagent (Sigma Aldrich, Steinheim, Germany). The quality and quantity of RNA and microRNA was estimated spectrophotometrically with BioPhotometer plus (Eppendorf, Hamburg, Germany). The sample was characterized with a A_260_ nm/A_280_ nm ratio, which was in the range of 1.70–2.00. cDNA synthesis for RNA was performed with High-Capacity cDNA Reverse Transcription Kit (Applied Biosystems, Waltham, MA, USA) in accordance with the manufacturer’s protocol. Next, cDNA were mixed with PowerUp SYBR Green Master Mix (Applied Biosystems, Waltham, MA, USA), forward primer 5′-ATGAAATCCCCAAGGAGAACC-3′ and reverse primer 5′-TTTCAAGCAGTTACTATCAATGA-3′, as well as RNase-free water in a total volume of 10 μL. Cycle parameters for the reaction were as follows: UDG activation at 50 °C for two minutes and Dual-Lock DNA polymerase at 95 °C for two minutes, followed by 40 cycles of sequential incubations at 95 °C for 15 s and at 60 °C for one minute. PCR products were verified during agarose gel electrophoresis. All experiments were performed in triplicate. The reaction was performed using a Mastercycler ep Realplex4s (Eppendorf, Hamburg, Germany). The RNA for positive control were obtained from the plasmid containing the cDNA of ADGRF5 and the RNA for negative control was obtained from untransfected HEK 293 cells (Supplementary Figure S2A).

#### 4.3.2. Immunocytochemistry

HEK-ADGRF5 cells were plated onto a 96-well plate at a confluence of 5 × 10^4^ cells per well. After 24 h cells were washed with phosphate-buffered saline (PBS) and blocked with FBS for 15 min at 4 °C. HEK-ADGRF5 cells were incubated for 1 h at 4 °C with the primary antibody against ADGRF5 (SAB4500874, (Sigma Aldrich) at 1:100 dilution). After the incubation, the primary antibody against ADGRF5 was removed and the cells were washed with PBS. Then, the cells were incubated with the AlexaFluor^®^ 488 [A32731, ThermoFisher Scientific (TFS), Waltham, MA, USA] goat anti-rabbit secondary antibodies for 40 min at 4 °C. Finally, the cells were washed with PBS and analyzed by fluorescence emission using Leica TCS SP8 confocal microscope (Leica-Microsystems, Wetzlar, Germany) (Supplementary Figure S2B).

### 4.4. Peptides

Peptides were obtained from Lipopharm (Gdansk, Poland) as TFA salts and dissolved as 2.5 mM stock solution in syringe-filtered (0.22 µm) 1 × HBSS with 20 mM HEPES (no Ca^2+^, no Mg^2+^, pH = 7.4). To note, the purity of peptides was evaluated using matrix-assisted laser desorption/ionization and liquid chromatography–mass spectrometry analysis.

### 4.5. Calcium Flux Assay

The evaluation of calcium flux was determined using FLIPR Calcium 6 Assay Kit obtained from Molecular Devices (San Jose, CA, USA) and a Biotek Synergy H1 plate reader equipped with a dual syringe injector (Winooski, VT, USA). ADGRF5-overexpressing or WT HEK-293 cells were seeded onto a black 96-well plate at a density of 50 × 10^6^ cells per well and allowed to attach overnight. On the next day, the cells were loaded with the calcium indicator for 2 h at 37 °C. Then, the baseline fluorescence signal was recorded (λ_Ex_ = 485 nm; λ_Em_ = 525 nm), followed by the injection of the peptides of interest or vehicle (1 × HBSS with 20 mM HEPES, no Ca^2+^, no Mg^2+^) and subsequent recording of the response to the treatment. To note, maximum concentration was restricted by both peptide solubility and assay volume constraints. All readouts were conducted in the atmosphere of 5% CO_2_ at a temperature of 37 °C. Upon background subtraction, a dose–response curve was plotted for each peptide and the area under the curve was calculated as a measure of activity.

### 4.6. Inositol Phosphate Accumulation Assay

The measurement of IP1 accumulation was carried out using IP-One ELISA kit from Cisbio Bioassays (Codolet, France), all according to the manufacturer’s instructions.

### 4.7. Protein Isolation and Western Blot

Proteins were isolated in the ice-cold 1 × Cell lysis buffer [#9803, Cell Signaling Technology (CST), Danvers, MA, USA] supplemented with 1 mM phenylmethane sulfonyl fluoride (PMSF, #93482, Sigma Aldrich) and 1 × protease and phosphatase inhibitor cocktail (#78442, TFS). The lysates were centrifugated for 10 min at 18,100× *g* at 4 °C. The total protein concentration in the obtained homogenates was evaluated in each sample in duplicates using BCA assay (#23225, TFS). Exactly 15 μg of protein samples were separated on 4–12% pre-cast polyacrylamide gels (#NW04127BOX, TFS) in MES-SDS electrophoresis buffer (#B002-02, TFS) and electrotransferred using iBlot2 system (TFS) onto polyvinyl difluoride (PVDF) membranes (#IB24001, TFS). Membranes were blocked for 30 minutes in 3% bovine serum albumin in 1 × TBST (tris-buffered saline with Tween 20; 20 mM tris, 150 mM NaCl, 0.1% Tween 20) before incubation with commercially available primary rabbit antibodies (dilution 1:1000) from CST against peEF2 (#2331), teEF2 (#2332), pAKT (#4060), tAKT (#4691), pERK1/2 (#4370), tERK1/2 (#4695), PKA p-substrates (#9624), PKC p-substrates (#6967), or AMPK p-substates (#5759) overnight at 4 °C. For each sample, a separate analysis was performed using rat α-tubulin (#MA180017, TFS) or rat β-actin (#664802, BioLegend, San Diego, CA, USA) antibodies (dilution 1:2000), which served as a loading control. Unbound antibodies were washed two times for 4 min and two times for 2 min with TBST. Subsequently, membranes were incubated with anti-rabbit secondary antibodies (dilution 1:5000) coupled with HRP (#7074, CST) or anti-rat secondaries conjugated with AlexaFluor 555 (#4417, CST) for 30 min at room temperature. Unbound secondary antibodies were washed using the same washing protocol as before. When necessary, the membranes were subjected to Westar Supernova substrate (Cyanagen, Bologna, Italy) for 2 min. Immunoreactive bands were visualized using Azure C400 imaging system (Azure Biosystems, Dublin, CA, USA) and quantified densitometrically using Fiji 1.53t image processing package [22].

### 4.8. Mice

Male C57/BL6NJ wild-type mice were bred in-house and 8-week-old mice weighing 25–30 g were used for all experiments. Mice were housed at a constant temperature (22–24 °C), humidity ~55% and maintained under a 12 h light/dark cycle with free access to laboratory chow and water ad libitum. Groups of 10–12 animals were in the experiment. The study were conducted in accordance with experimental protocol approved by the Local Ethical Committee at the Medical University of Lodz (Lodz, Poland) and completed with European Communities Council Directive of 22 September 2010 (2010/63/EU).

### 4.9. Murine Model of Colitis and Macroscopic Analysis of Colons

Murine model of colitis was induced by the addition of 2.5% dextran sodium sulfate (DSS) slat to drinking autoclaved water from day 0 to day 5. On day 6, animals received autoclaved water without DSS. To note, control animals received autoclaved water throughout the entire experiment. PL01^WT^, PL24^S11N,D13H^, and PS31^S11N^ peptides were dissolved in phosphate-buffered saline (PBS) and were injected once a day intraperitoneally (i.p.) at doses of 1 mg/kg or 5 mg/kg to a final volume of 100 µL from day 1 to 5. Animals without treatment received 100 µL of PBS. Animal health and body weight were monitored daily. On day 8, mice were sacrificed through rapid dislocation of the cervical vertebrae and the evaluation of colonic damage were performed. The total macroscopic damage score were estimated based on the following parameters: presence of diarrhea (0–3), the extent of colon damage (0–3), colon length (0–4; 0 = below 5% shortening; 1 = 5–14% shortening, 2 = 15–24% shortening, 3 = 25–35% shortening, 4 = more than 35% shortening as compared to control group) and the colon weight (0–4; 0 ≤ 5%; 1 = 5–14%; 2 = 15–24%; 3 = 25–35%, 4 = more than 35% as compared to control group). The presence (score = 1) or absence (score = 0) of fecal blood was also included into the macroscopic damage score. Further, colons from male mice were washed in PBS and collected for histological staining and microscopic analysis.

### 4.10. Microscopic Score Evaluation

Colon samples were rolled into *Swiss rolls* and fixed in 10% formalin, dehydrated, and embedded in paraffin. The 5 µm sections were sectioned using Leica RM2255 fully automated rotary microtome (Leica-Microsystems, Germany) and mounted onto slides. The sections were stained hematoxylin and eosin (Sigma Aldrich) and examined. Photographs were taken using hardware (AX-70 light microscope, Imaging Solutions Camera, Fairport, NY, USA) and software (Cell F Imaging Software, version 2.5) from Olympus and Olympus Soft Imaging Solutions (Münster, Germany), respectively. Microscopic total damage score was determined based on the following parameters: destruction of the mucosal layer (normal = 1, moderate = 2, extensive = 3), the goblet cell depletion (presence = 1, absence = 0), crypt abscesses (presence = 1, absence = 0), the extent of muscle thickening (normal = 1, moderate = 2, extensive = 3), and the presence and degree of cellular infiltration (normal = 1, moderate = 2, transmural = 3).

### 4.11. Statistical Analysis

Statistical analysis was performed using GraphPad Prism 8.0 (GraphPad Software, San Diego, CA, USA). Results are presented as means ± standard error of the mean (SEM). Half-maximal effective concentrations (EC_50_) were calculated by fitting the control-normalized experimental data to a four-parameter logistic equation. One-way ANOVA followed by a Dunnett post hoc test was to compare the studied groups. *p*-values < 0.05 were considered statistically significant.

## Figures and Tables

**Table 1:** The list of peptide ADGRF5 agonists with indicated mutations introduced into the *Stachel* sequence. The list includes longer 13-amino acid peptides (PL series) and shorter 12-amino acid peptides (PS series).

Peptide ID	Peptide Sequence	Type of Changes	Peptide Length
NCP	TSFSILMSSKPVK	-	13
PL01^WT^	TSFSILMSPDSPD	-	13
PL02^P9A^	TSFSILMSADSPD	position 9 P → A	13
PL03^P12A^	TSFSILMSPDSAD	position 12 P → A	13
PL04^S8T^	TSFSILMTPDSPD	position 8 S → T	13
PL05^S8C^	TSFSILMCPDSPD	position 8 S → C	13
PL06^D10E^	TSFSILMSPESPD	position 10 D → E	13
PL07^D10N^	TSFSILMSPNSPD	position 10 D → N	13
PL08^S11T^	TSFSILMSPDTPD	position 11 S → T	13
PL09^S11C^	TSFSILMSPDCPD	position 11 S → C	13
PL10^D13E^	TSFSILMSPDSPE	position 13 D → E	13
PL11^D13N^	TSFSILMSPDSPN	position 13 D → N	13
PL12^S11M^	TSFSILMSPDMPD	position 11 S → M	13
PL13^S11H^	TSFSILMSPDHPD	position 11 S → H	13
PL14^S11N^	TSFSILMSPDNPD	position 11 S → N	13
PL15^D13Q^	TSFSILMSPDSPQ	position 13 D → Q	13
PL16^D13H^	TSFSILMSPDSPH	position 13 D → H	13
PL17^D13S^	TSFSILMSPDSPS	position 13 D → S	13
PL18^S11T,D13E^	TSFSILMSPDTPE	positions 11 S → Tposition 13 D → E	13
PL19^S11T,D13N^	TSFSILMSPDTPN	position 11 S → Tposition 13 D → N	13
PL20^S11C,D13E^	TSFSILMSPDCPE	position 11 S → Cposition 13 D → E	13
PL21^S11C,D13N^	TSFSILMSPDCPN	position 11 S → Cposition 13 D → N	13
PL22^S11N,D13S^	TSFSILMSPDNPS	position 11 S → Nposition 13 D → S	13
PL23^S11T,D13S^	TSFSILMSPDTPS	position 11 S → Tposition 13 D → S	13
PL24^S11N,D13H^	TSFSILMSPDNPH	position 11 S → Nposition 13 D → H	13
PL25^S11T,D13H^	TSFSILMSPDTPH	position 11 S → Tposition 13 D → H	13
PS26^WT^	TSFSILMSPDSP	-	12
PS27^S11T^	TSFSILMSPDTP	position 11 S → T	12
PS28^S11C^	TSFSILMSPDCP	position 11 S → C	12
PS29^S11M^	TSFSILMSPDMP	position 11 S → M	12
PS30^S11H^	TSFSILMSPDHP	position 11 S → H	12
PS31^S11N^	TSFSILMSPDNP	position 11 S → N	12
PS32^S11Y^	TSFSILMSPDYP	position 11 S → Y	12

## Data Availability

The original contributions presented in this study are included in the article/Supplementary Material. Further inquiries can be directed to the corresponding author.

## Supplementary Materials

The following supporting information can be downloaded at https://www.mdpi.com/article/10.3390/ijms27062648/s1.

## Abbreviations

The following abbreviations are used in this manuscript:

7TM	7-transmembrane
aa	amino acids
ADGRF5	adhesion G protein-coupled receptor F5
AF3	AlphaFold3
AKT	Ak strain transforming
AMPK	5′-AMP-activated protein kinase
CD	Crohn’s disease
DSS	dextran sulfate sodium
EC_50_	half-maximal effective concentration
eEF2	eukaryotic elongation factor 2
ERK1/2	extracellular signal-regulated kinases 1/2
GAIN	GPCR autoproteolysis-inducing
GPCR	G protein-coupled receptor
GPR116	G protein-coupled receptor 116
GPS	GPCR proteolysis site
IBD	inflammatory bowel diseases
i.p.	intraperitoneally
IP	inositol phosphate
NCP	negative control peptide
PKA	protein kinase A
PKC	protein kinase C
pLDDT	per-residue confidence prediction
pTM	predicted template modeling
SEA	sea urchin sperm protein, Enterokinase and Agrin
TM	transmembrane
UC	ulcerative colitis
