# Supplementary material for: Anti-Inflammatory Potential of Novel Tethered Agonists of the Adhesion G Protein-Coupled Receptor F5
Source: Int J Mol Sci. 2026 Mar 13;27(6):2648. doi: 10.3390/ijms27062648 (PMC13026237; doi:10.3390/ijms27062648)
Supplement: Supplementary file 1 [file ijms-27-02648-s001.zip › ijms-4155714-supplementary.pdf]

Anti-inflammatory potential of novel tethered agonists of the adhesion G protein-coupled receptor F5

Artur Wnorowski[1], Diana Pietrzak[1], Akanksha Mudgal[1], Lorenzo Scrofani[1,2], Magdalena Strachowska[3], Piotr Draczkowski[4,5], Krzysztof Jóźwiak[1], Jakub Fichna[6], Damian Jacenik[3,#]

1 – Department of Biopharmacy, Faculty of Pharmacy, Medical University of Lublin, Lublin, Poland

2 – Department of Pharmacy and Biotechnology, University of Bologna, Bologna, Italy

3 – Department of Cytobiochemistry, Faculty of Biology and Environmental Protection, University of Lodz, Lodz, Poland

4 – Department of Synthesis and Chemical Technology of Pharmaceutical Substances, Faculty of Pharmacy, Medical University of Lublin, Lublin, Poland

5 – Department of Biochemistry and Biophysics, National Bioinformatics Infrastructure Sweden, Science for Life Laboratory, Stockholm University, Solna, Sweden

6 – Department of Biochemistry, Faculty of Medicine, Medical University of Lodz, Lodz, Poland

# – correspondence: Damian Jacenik, 141/143 Pomorska St., 90-236 Lodz, Poland, e-mail: damian.jacenik@biol.uni.lodz.pl

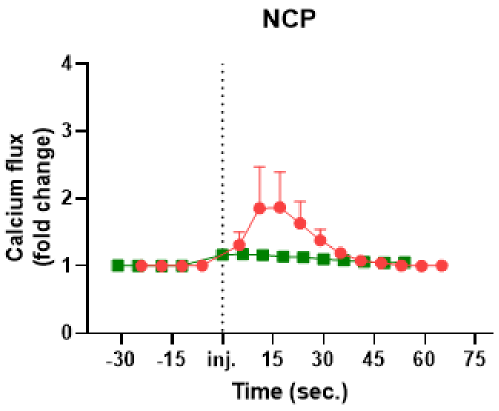

Figure S1. Activity of mature tethered ADGRF4 agonist (NCP) were estimated in HEK-293 cells with ADGRF5 overexpression using calcium flux. Data are expressed as mean ± SEM, n = 3 for independent experiments and n = 3 – 4 for technical replicates.

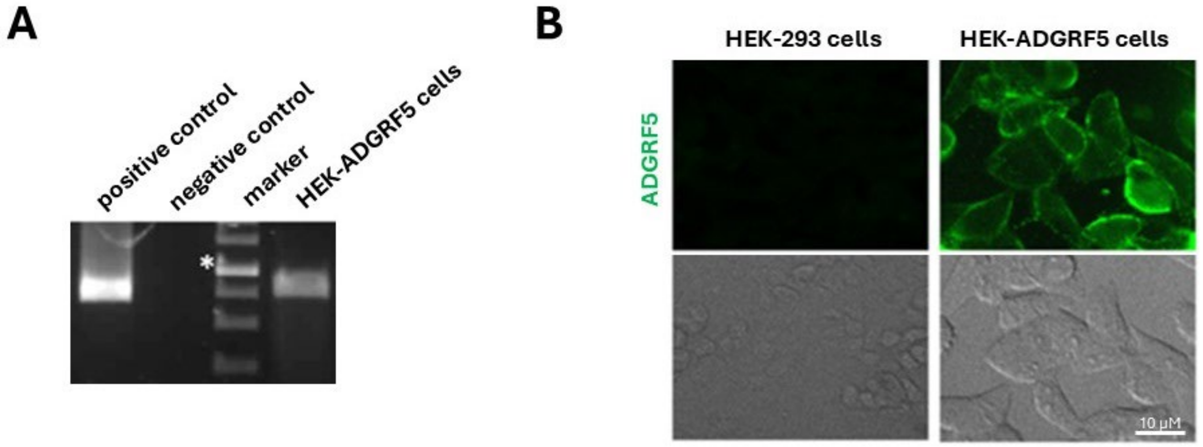

Figure S2. Representative image of ADGRF5 expression at the mRNA level (**A**) in HEK-293 cells (negative control) and HEK-293 cells with ADGRF5 overexpression (HEK-ADGRF5 cells). To note, human ADGRF5 cDNA as a positive control was used. Representative images of immunofluorescence staining of ADGRF5 (**B**) in HEK-293 cells and HEK-ADGRF5 cells.
